# Hemolytic Anemia Secondary to Rasburicase Treatment in the Setting of Glucose-6-Phosphate-Dehydrogenase Deficiency: A Case Report

**DOI:** 10.7759/cureus.72024

**Published:** 2024-10-21

**Authors:** Jacob C Stone, Sharon Santhosh, Awais Paracha, Brittany Kwait, Veena John

**Affiliations:** 1 Internal Medicine, Northwell Health, New Hyde Park, USA; 2 Hematology and Medical Oncology, Northwell Health, New Hyde Park, USA

**Keywords:** drug-induced hemolysis, drug-induced hemolytic anemia, glucose-6-phosphate-dehydrogenase deficiency (g6pdd), glucose-6-phosphate dehydrogenase (g6pd), rasburicase

## Abstract

Rasburicase is a recombinant form of urate oxidase, a medication used to treat hyperuricemia by metabolizing uric acid into an inactive and more soluble metabolite, allantoin. An oxidizing agent, hydrogen peroxide, is produced during the conversion of uric acid to allantoin. We present here a case of hemolytic anemia secondary to rasburicase treatment which was later confirmed to have glucose-6-phosphate dehydrogenase deficiency (G6PDD). Treatment was stopped after one dose and the patient received a transfusion of packed red blood cells to correct the anemia. The patient’s hyperuricemia was subsequently managed with allopurinol and febuxostat.

## Introduction

Glucose-6-phosphate dehydrogenase (G6PD) is a cytosolic enzyme that replenishes the supply of intracellular nicotinamide adenine dinucleotide phosphate (NADPH). NADPH in turn replenishes glutathione, a molecule that neutralizes reactive oxygen species to prevent cellular damage. G6PD deficiency (G6PDD) is a group of inherited deficiencies in activity of G6PD. Individuals with G6PDD are therefore at increased risk of oxidative damage [1]. Here, we report a patient who developed hemolytic anemia likely due to rasburicase treatment, in whom subsequent testing revealed low serum levels of G6PD function. 

## Case presentation

**Figure 1:**
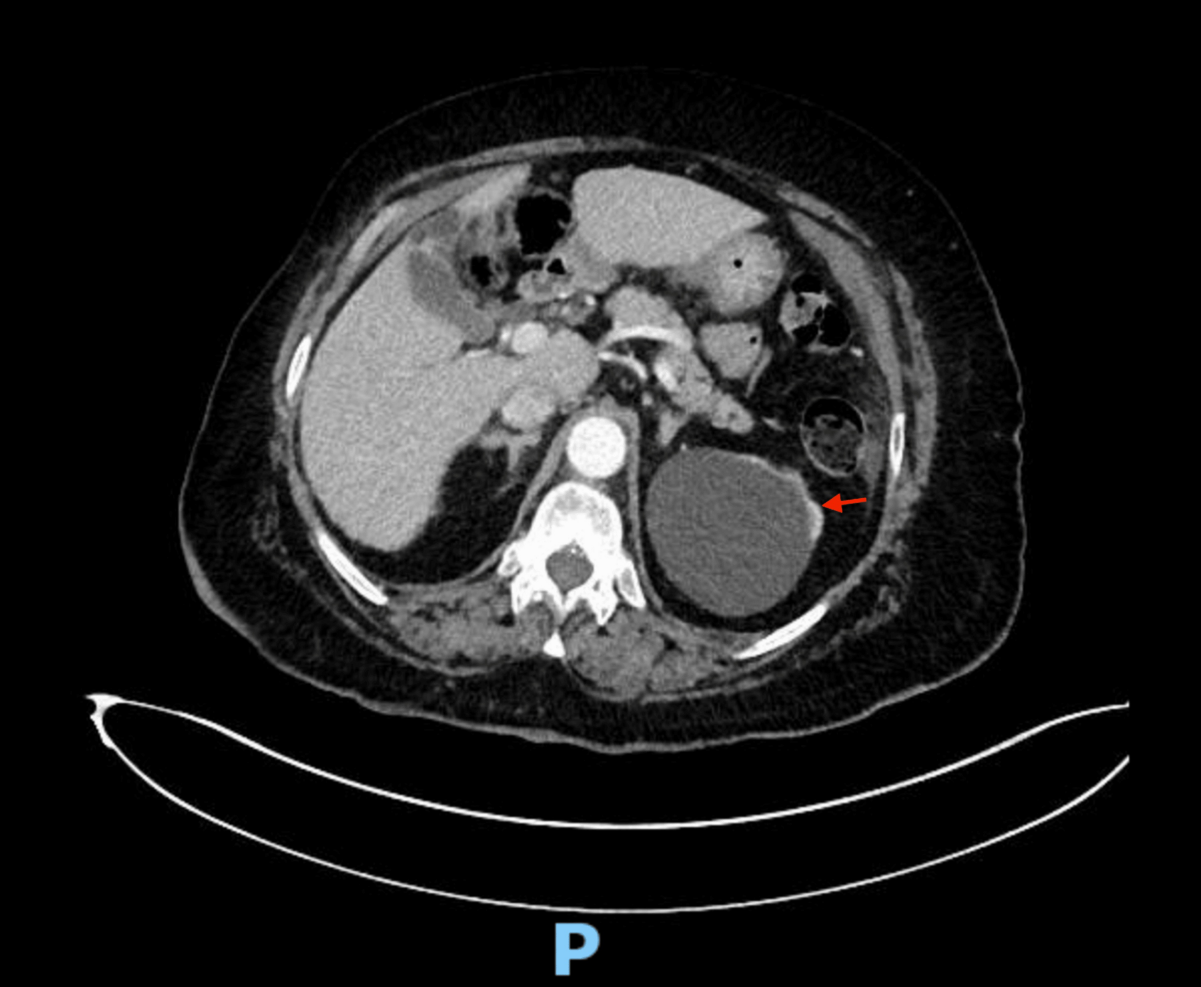
CT abdomen revealing a lower left quadrant 7.5 x 8 cm soft tissue mass. Red arrow indicates the mass.

An 88-year-old African American woman with a past medical history of follicular lymphoma in 2010 (treated with Rituxan) and Hodgkin lymphoma in 2018 (treated with brentuximab vedotin, doxorubicin, vinblastine, and dacarbazine) presented to our hospital in May 2024 with confusion, right-sided weakness, and slurred speech. Imaging revealed a left lower quadrant soft tissue mass measuring 7.5 x 8 cm, mesenteric and retroperitoneal lymphadenopathy, and small-volume ascites (Figure 1). MR angiogram of the head did not demonstrate acute intracranial hemorrhage or acute ischemia, although there was evidence of multiple chronic infarcts. Upon laboratory workup, she was found to have a serum creatinine of 1.48 mg/dL and a serum calcium level of 13.3 mg/dL. Further testing revealed a serum uric acid level of 9.8 mg/dL.

The patient was treated with 3 mg rasburicase intravenously for the management of her hyperuricemia. The patient reported no history of rasburicase treatment, family history of anemia, or family or personal history of hemolysis. Three days after treatment with rasburicase, the patient was found to have a hemoglobin level of 6.9 g/dL, down from 9.5 g/dL prior to rasburicase administration, an indirect bilirubin level of 2.8 mg/dL, a serum haptoglobin of <20 mg/dL, and a reticulocyte percent of 4.3%, indicating new hemolytic anemia. The patient’s baseline total bilirubin, measured before rasburicase administration, was 0.4 mg/dL. 

**Figure 2:**
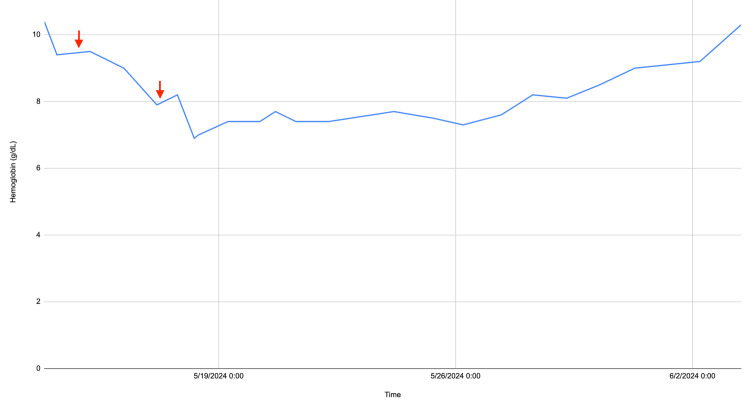
Patient’s hemoglobin level (g/dL) over the course of her hospital stay. First red arrow indicates timing of administration of rasburicase. Second red arrow indicates timing of administration of packed red blood cells.

Rasburicase was discontinued and the patient received a transfusion of packed red blood cells for correction of her anemia. Levels of serum G6PD had been drawn before administration of the rasburicase, and resulted after to be 4.6 U/g Hgb (normal: 7.0-20.5 U/g Hgb), indicating G6PDD. The patient was then switched to allopurinol and febuxostat for treatment of her hyperuricemia. The hemolytic anemia continued to resolve, with normalization of serum haptoglobin and bilirubin to the patient’s baseline levels ten days following treatment with rasburicase. The patient's hemoglobin levels over the course of her hospital stay are provided in Figure 2.

A biopsy of the patient’s lower left quadrant mass revealed diffuse large B-cell lymphoma (DLBCL). Given fluorescence in situ hybridization (FISH) results showing an IGH/BCL2 fusion, which is commonly seen in follicular lymphoma, the DLBCL was considered to likely be a transformation from the patient’s previous follicular lymphoma. The DLBCL was therefore treated with rituximab, loncastuximab, and a five-day course of steroids. She was ultimately discharged from our hospital with plans to continue outpatient treatment.

Outpatient, she was continued on a regimen of loncastuximab every three weeks until September 2024, for a total of five cycles. At that time she was switched to a regimen of rituximab, cyclophosphamide, vincristine, and prednisolone due to the progression of her disease.

## Discussion

G6PDD is the most prevalent enzyme deficiency, with an estimated 400 million people affected worldwide [2]. Patients with G6PDD are unable to replenish intracellular glutathione levels, which makes them susceptible to hemolytic anemia in the setting of oxidative stress. Red blood cells carry oxygen throughout the body and are unable to replace oxidatively damaged proteins because they lack a nucleus, so they are the most susceptible to the effects of oxidative damage in G6PDD [3]. As rasburicase generates hydrogen peroxide in its conversion of uric acid to allantoin, it increases the oxidative stress placed upon the body and can cause hemolytic anemia in patients with G6PDD [4].

Hemolysis usually begins 24 to 72 hours after exposure to the causative agent, with resolution within four to seven days [5]. This clinical course is consistent with the timeline of our patient’s episode of hemolysis.

Patients being considered for rasburicase treatment who are at high risk of G6PDD, such as those with a history of prior drug-induced hemolytic anemia or individuals of African, Asian, and Mediterranean descent, especially males, should receive testing for levels of G6PD activity before receiving therapy [4]. In some populations, such as Black males in the United States, the prevalence of G6PDD can be as high as 10%, so extra care should be taken before administering oxidative medications to patients from these populations [5]. Males are more susceptible to severe disease as the locus for the G6PD protein is on the X chromosome. Thus, males who inherit a diseased allele have severely reduced G6PD function.

Females who are carriers for G6PDD may have a mosaic expression of the G6PDD phenotype. Due to the inactivation of one of the X chromosomes in female development, only some red blood cells produced in female heterozygotes are deficient in G6PD function. The degree of G6PD deficiency is influenced by the proportion of red blood cells that originate from cell lines in which X-inactivation silenced the chromosome containing the normal gene in development [6]. Since X-inactivation is a random process, the quantitative amount of G6PD activity in heterozygous females is not always exactly 50% of normal. Overall levels of G6PD activity in heterozygous females mainly range from 30-80% of normal [7]. And when an oxidative crisis occurs, the symptoms are typically not as severe as those in male patients as some red blood cells are unaffected [8].

It is possible that this female patient was heterozygous for G6PDD. While the quantitative results of this patient’s G6PD activity levels are consistent with moderately reduced G6PD function (4.6 U/g Hgb), no genetic testing was performed to confirm heterozygosity. This patient represents a population that presents a clinical challenge, as some heterozygous patients may have episodes of hemolysis in situations of severe oxidative stress even though most heterozygotes usually do not express clinical symptoms of disease.

Overall, few studies have looked at drug safety and the incidence of adverse effects such as hemolysis in females with moderately reduced levels of G6PD function, such as our patient [7]. In one study of female heterozygotes treated with primaquine, another oxidative medication, higher doses were found to correlate with a higher likelihood of clinically significant hemolysis, with two heterozygous females requiring blood transfusion [9]. Additionally, other case reports have also identified females with moderately reduced G6PD function with clinically significant methemoglobinemia and hemolysis, similar to our patient [10].

## Conclusions

Patients receiving treatment with rasburicase should be tested for levels of G6PD activity before receiving treatment and should be monitored for signs of hemolysis following treatment. More research is necessary to determine the frequency and severity of adverse effects of oxidative medications in female patients who have moderately reduced G6PD function, who are often heterozygous for G6PDD.
